# Donor DNA: Embedding self-regulation into blood donation culture

**DOI:** 10.1016/j.htct.2025.106241

**Published:** 2026-01-10

**Authors:** Dr RishiRaj Sinha

**Affiliations:** Department of Transfusion Medicine, All India Institute of Medical Sciences (AIIMS), New Delhi, India

Dear Editor,

I read with great interest the article by Ranjbar Kermani et al. on the development and validation of the Self-Regulation of Blood Donation Scale (SRBDS) for Iranian blood donors [1]. This pioneering work adds depth to our understanding of donor motivation by contextualizing it within the self-determination theory (SDT) framework. While the scale demonstrates strong psychometric properties, I believe its implications extend far beyond measurement. In this correspondence, I propose several novel avenues to translate self-regulation science into actionable strategies for donor recruitment and retention.

1. **Digital Behavioural Nudges and Gamification**
  The SRBDS highlights intrinsic and integrated regulation as key drivers of sustained donation [1]. Embedding these dimensions into digital blood donor apps could transform engagement. For instance, gamified features that reward streaks of consistent donation, peer challenges, and personalized motivational feedback could amplify intrinsic satisfaction, as supported by SDT research in other health behaviors [2].
2. **Culturally Adapted Messaging Beyond Iran**
  The study reflects the importance of religious and cultural motivations in Iran [1]. However, cross-cultural adaptation is vital. In Western settings, altruism and community solidarity dominate donor narratives, while in parts of Asia, familial duty or reciprocity may prevail [3]. A modular, culturally sensitive adaptation of the SRBDS could help global blood services tailor campaigns to local motivational landscapes.
3. **Integrating Psychometrics into Donor Management Systems**
  Routine use of the SRBDS during donor registration could generate valuable motivational profiles. Linking these profiles with donor return data in electronic donor management systems would allow predictive analytics to identify ‘at-risk’ lapsed donors and target them with personalized interventions [4]. This approach shifts from reactive donor recruitment to proactive donor relationship management.
4. **Motivational Interviewing as a Retention Tool**
  Evidence from other domains suggests motivational interviewing enhances internalization of health behaviors [5]. Training donor recruiters to use brief motivational interviewing techniques, rooted in SRBDS dimensions, could reinforce identified and integrated regulation, thereby promoting long-term donor identity formation.
5. **Research on Motivation Transitions Over Time**
  While the SRBDS validation was cross-sectional, longitudinal research could uncover how motivations evolve with donor experience. Do donors shift from external to intrinsic regulation as they internalize a “donor identity”? Understanding these transitions could inform stage-specific retention strategies, from first-time donor encouragement to legacy donor recognition.

In conclusion, the SRBDS represents more than a psychometric advance; it is a roadmap to reimagine donor retention strategies grounded in behavioral science. By embedding self-regulation principles into digital tools, cultural tailoring, donor management systems, and motivational interviewing, transfusion services can transform donor motivation into mobilization.

## Data availability statement

The data that support the findings of this study are available from the corresponding author upon reasonable request.

## Conflicts of interest

There are no conflicts of interest.

## References

[1] Ranjbar Kermani F., Amini Kafi-Abad S., Maghsudlu M., Hosseini K.M., Mohammadali F., MohammadJafari A. (2024). Development and validation of the self-regulation of blood donation scale for blood donors. Hematol Transfus Cell Ther.

[2] Ryan R.M., Deci E.L. (2000). Self-determination theory and the facilitation of intrinsic motivation, social development, and well-being. Am Psychol.

[3] Maghsudlu M., Nasizadeh S. (2011). Iranian blood donors’ motivations and their influencing factors. Transfus Med.

[4] Romero-Domínguez L., Martín-Santana J.D., Sánchez-Medina A.J., Beerli-Palacio A. (2021). Lines of scientific research in the study of blood donor behavior from a social marketing perspective. J Nonprofit Public Sect Market.

[5] Livitz I.E., Fox K.R., Himawan L.K., France C.R. (2017). A brief motivational interview promotes internal motivation to donate blood among young adults. Transfusion (Paris).

